# Efficiency of the intestinal bacteria in the degradation of the toxic pesticide, chlorpyrifos

**DOI:** 10.1007/s13205-012-0078-0

**Published:** 2012-08-01

**Authors:** M. K. Harishankar, C. Sasikala, M. Ramya

**Affiliations:** Department of Genetic Engineering, SRM University, Chennai, 603203 Tamil Nadu India

**Keywords:** Chlorpyrifos, Biodegradation, Diethylthiophosphate, Organophosphorous phosphatase

## Abstract

Chlorpyrifos (CP) is the most commonly used pesticide throughout the world. Its widespread use in agriculture and its potential toxicity to humans from ingestion of CP contaminated food have raised concerns about its risk to health. Human intestinal microflora has the ability to degrade pesticides, but the exact mechanisms involved and the metabolite end-products formed are not well understood. The primary objective of this work was to analyse the in vitro degradation of CP by five model intestinal bacteria namely *Lactobacillus lactis*, *L. fermentum*, *L. plantarum*, *Escherichia coli* and *Enterococcus faecalis*. Plate assay results revealed that *L. lactis*, *E. coli* and *L. fermentum* could grow with high concentrations of CP (>1,400 μg/mL), whereas *E. faecalis* and *L. plantarum* could grow with concentrations as low as 400 and 100 μg/mL, respectively. The best three CP degraders were therefore used in further experiments. The degradation of CP-induced organophosphorous phosphatase (OPP) production and that OPP concentration were higher in the supernatant (extracellular) rather than inside the cells by factor of up to 28. *L. fermentum* degraded 70 % CP with 3,5,6-trichloro-2-pyridinol (TCP) detected as the end product. *L.lactis* degraded up to 61 % CP with chlorpyrifos oxon detected as the end product, whereas *E.coli* degraded a lesser concentration (16 %) to chlorpyrifos-oxon and diethylphosphate.

## Introduction

Chlorpyrifos [*O*,*O*-diethyl *O*-(3,5,6-trichloro-2-pyridinyl) phosphorothioate] (CP) is one of the most commonly used agricultural organophosphorous (OP) insecticides, which controls a broad spectrum of insects. Despite the recent regulatory decision of the United States to eliminate its residential use, CP continues to be widely used in agriculture in other regions of the world including Egypt, Germany, China, India, Bangladesh, Pakistan, and Iraq. CP acts by interfering with cholinesterase, an enzyme that is essential for the proper working of the nervous system of both humans and insects (Xu et al. 2008). The widespread use of CP in agriculture has raised public concerns about the potential human health risks that can be caused by the ingestion of CP-contaminated foods (Atif Randhawa et al. 2007). In general, CP can enter the human body through the skin (dermal exposure), mouth (oral exposure) and lungs (respiratory exposure). However, the transport of CP within the body depends on whether it is absorbed through the skin, lungs or gastrointestinal (GI) tract. CP absorbed through the GI tract, enters the blood stream and reaches the liver, the major site of pesticide metabolism, resulting in liver toxicity. Moreover, CP can also be accumulated in the body tissues, proteins, fats and bones for longer period of time causing additional health hazards (Environmental Risk assessment 2002). CP is degraded in soil and aquatic environment by chemical hydrolysis and by microbial activities. However, the rate of degradation by chemical hydrolysis is very low when compared to microbial degradation, and this could be attributed to the presence of efficient hydrolytic and oxidative enzymes which degrade these xenobiotic compounds (Munnecke 1976). The accessibility of the pesticide to the cell across the cell membrane is one of the most important considerations for degradation. Degradation of different pesticides can range from high to low because of accessibility issues. Hence, it is essential to study the location and function of the enzyme to understand its significance in degradation of studies. (Richnis et al. (1997) studied the surface-expressed organophosphorous hydrolase activity on the organophosphorous pesticide and found that more than 80 % of the activity was located on cell surface, but in case of *Nocardiodes simplex* NRRL B-24074 it was located as a distinct enzyme in the cytoplasm (Mulbry 2000). In most cases, aerobic bacteria tend to transform CP to produce diethylthiophosphate (DETP) and 3,5,6-trichloro-2-pyridinol (TCP) (Yang et al. 2006). However, the isolation and detailed studies of CP degrading bacteria have been difficult because TCP, the hydrolytic by-product of CP, is toxic to the growth of the degrading organism. The bacterium capable of using TCP as the sole carbon and energy source under aerobic conditions was identified as a *Pseudomonas* sp. (ATCC 700113), by Feng et al. (1997). Subsequently, other CP degrading bacteria such as *Ralstonia* sp. (Li et al. 2010), *Lactobacillus brevis* (Islam et al. 2009), *Bacillus pumilus* strain (Anwar et al. 2009), *Pseudomonas aeruginosa*, *Bacillus cereus*, *Klebsiella* sp. (Lakshmi et al. 2009), *Paracoccus* sp. (Xu et al. 2008) and *Sphingomonas* sp strain DSP-2 (Li et al. 2007) have been reported. *L. lactis*, *L. plantarum*, *L. fermentum*, *E. faecalis* and *E.coli* are intestinal bacteria which are reported to prevent major intestinal infections (Biller et al. 1995) and are involved in the alleviation of inflammatory bowel disease (Sartor 2004), production of antimicrobial substances (Servin 2004) and regulation of gastrointestinal immunity(Christensen et al. 2002). Along with this, it was reported by Zhao and Wang (2011) that *Lactobacillus* sp. can also degrade several organophosphorous pesticides like dimethoate, fenthion, malathion, methyl parathion, monocrotophos, phorate and trichlorphon but its action on CP has yet to be studied. Moreover, the role of CP degrading microflora and the site of CP degradation in the GI tract are also poorly understood (Rose et al. 2005). Here, we report on the degradation of CP by five model intestinal bacteria which included *L. lactis*, *L. plantarum*, *L. fermentum*, *E.faecalis* and *E.coli.*

## Materials and methods

### Pesticide and chemicals

Commercial-grade insecticide chlorpyrifos (50 % E.C) was purchased from Dow Agro Sciences, India Private Limited. Chlorpyrifos and 3,5,6-trichloro-2-pyridinol (TCP) were purchased from Sigma-Aldrich Co., USA. All other chemicals and media used in this study were purchased from Hi-Media Private Ltd, Mumbai, India.

### Bacterial strains and media

*Enterococcus faecalis* (MTCC 2729), *E. coli* (MTCC 433), *L. fermentum* (MTCC 903), *L. lactis* (MTCC 4185), *L. plantarum* (MTCC 1325) were purchased from Microbial Type Culture Collection (MTCC) centre, Chandigarh, India. The strains were stored in Luria–Bertani (LB) medium containing 20 % glycerol at −20 °C. LB Agar was used for routine culturing of the strains. Chlorpyrifos degradation analyses were carried out in Minimal Salt broth (MS broth). MS broth contained (g/L) Yeast Extract 1, K_2_HPO_4_ 1.5, KH_2_PO_4_ 0.5 g, (NH_4_)_2_SO_4_ 0.5, NaCl 0.5, MgSO_4_ 0.2, CaCl_2_ 0.05, FeSO_4_ 0.02. 100 mg/L of chlorpyrifos was filter sterilized and used for degradation experiments (Yang et al. 2006).

### Plate assay for chlorpyrifos

The maximum concentration of CP tolerated by the bacterial strains was determined by streaking the strains on MS agar plates containing various concentrations of chlorpyrifos 100–2,000 mg/L. All the plates were incubated for 37 °C until visible growth was observed (Shafiani and Malik 2003).

### Extraction of crude enzyme and organophosphorous phosphatase (OPP) assay

The cells grown in MS broth containing 100 mg/L chlorpyrifos were harvested and pelleted by centrifugation at 8,000 rpm for 10 min. The supernatant was used to determine extracellular OPP activity. The cell pellet was resuspended in 50 mmol/L Tris–HCl (pH 8) buffer containing 0.1 mmol/L phenylmethylsulfonyl fluoride (PMSF) and sonicated for 10 times, each for 10 s duration with 15 s incubation on ice between sonication, using a Digital Sonifier (Bandelin Electronics, Berlin, Germany). The lysate was centrifuged at 10,000 rpm for 30 min and the supernatant was used to determine intracellular OPP activity (Wang et al. 2008). Protein concentrations were determined by the method of Lowry et al. (1951). All the experiments were repeated three times. OPP activity was measured by adding 100 μL of crude enzyme to 900 μL of Tris HCl (pH 9) containing 10 mg/mL *p*-nitrophenol phosphate and the mixture was incubated for 10 min at 37 °C. The reaction was terminated by addition of 1 mL of 10 % trichloroacetic acid and 1 mL of 10 % Na_2_CO_3_ and the liberated yellow coloured end product *p*-nitrophenol was measured in a Ultrospec™ 2100 *pro* spectrophotometer (GE Healthcare) at 410 nm. One unit (U) of OPP activity is defined as the amount of enzyme liberating 1 μmol of *p*-nitrophenol per minute at 37 °C (Alvarez-Macarie et al. 1999).

### Degradation analysis by LCMS

Mid log phase culture (7 × 10^6^) of the three strains was inoculated into MS broth containing 100 mg/L chlorpyrifos and incubated in a shaking incubator at 37 °C (Uninoculated MS broth containing 100 mg/L chlorpyrifos was used as a control). After 15 days of incubation, the optical densities of the cultures were measured (OD 610 nm). 50 mL culture aliquots and 50 mL uninoculated control medium aliquots were centrifuged at 7,000 rpm for 10 min and the supernatant was used for degradation analysis. For this, the supernatant was extracted with an equal volume of dichloromethane and the bottom organic layer was aspirated and dried at room temperature. The residue was then dissolved in HPLC grade acetonitrile and analysed using liquid chromatography–mass spectroscopy (LC–MS) (Prominence, SHIMADZU) (Anwar et al. 2009). The LC–MS was equipped with an inertsil ODS3 column (50 × 3 mm) and a UV detector. The cartridges were conditioned with acetonitrile and washed with deionized water containing 0.1 % formic acid. Sample injection volume was 10 μL and a gradient mobile phase containing acetonitrile and 0.1 % formic acid in water was used at a flow rate of 0.2 mL/min. The oven temperature was maintained at 37 °C and the UV detector at 230 nm. Under these conditions, the retention time for chlorpyrifos was 18.6 min. Mass spectroscopy (MS) was performed using a Finnegan model MS (Thermo electron corporation, USA). The ion trap detector with atomic pressure chemical ionization (APCI) source was used for quantification in positive ionization mode. The operating conditions were APCI source: spray voltage (kV)—5.02, capillary voltage (V) −16.96, capillary temperature (°C)—275, capillary temperature (°C)—270. Ion detection system: dynode (kV)—14.86, multiplier (V)—821.2.

## Results and discussion

### Plate assay and strain selection

**Fig. 1:**
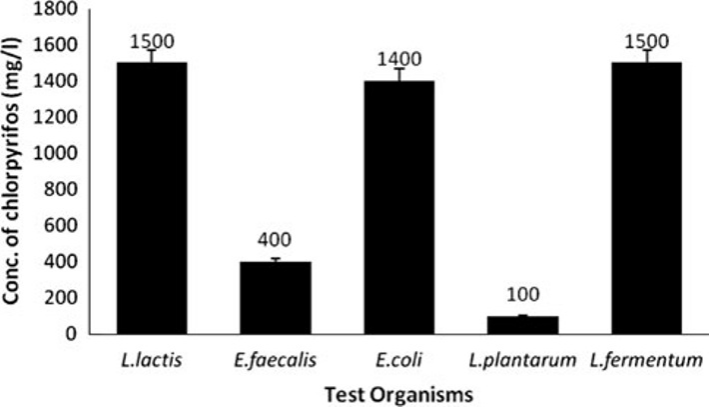
*Bar graph* showing growth of the selected bacterial strains on Minimal Salt (MS) agar plates supplemented with various concentrations (100–2,000 mg/L) of chlorpyrifos (CP). *L. lactis*, *E. faecalis*, *E. coli*, *L. plantarum* and *L. fermentum* were able to tolerate chlorpyrifos up to 1,500, 400, 1,400, 100 and 1,500 mg/L, respectively

Plate assays revealed that *L. fermentum*, *L. lactis* and *E. coli* had a similar higher tolerance to CP (>1,400 μg/mL) and the least tolerance was noted for *E. faecalis* (400 μg/mL) and *L. plantarum* (100 μg/mL) (Fig. 1). The variation in tolerance levels to different pesticides and pesticide concentrations is well documented. For example, Kale et al. (1989) reported that the growth of *Azotobacter chroococcum* was not affected carbofuran concentrations of up to 5 ppm but higher concentrations inhibited growth. Shafiani and Malik (2003) reported that a *Pseudomonas* sp. isolated from soil could tolerate up to 800 μg/mL of endosulfan, 1,600 μg/mL of carbofuran and 1,600 μg/mL of malathion. The three most tolerate strains, namely, *L. fermentum*, *L. lactis* and *E. coli* were used in further experiments.

### Organophosphorous phosphatase (OPP) assay

The enzyme organophosphorous phosphatase determined by the OPP assay was found to be present in both the intracellular as well as the extracellular fractions of the three selected bacteria, namely *L. fermentum*, *L. lactis* and *E. coli*. The activity of the extracellular fraction was consistently higher than the intracellular fraction (between 8 and 30-fold) (Table 1). Interestingly, the extracellular OPP activity was in the order *E. coli* > *L. lactis* > *L. fermentum* whereas this trend was reversed for intracellular activity with the order being *L. fermentum* > *L. lactis* > *E. coli.* All the three strains had negligible enzyme activity in uninduced CP lacking control cultures and confirming that the OPP was an inducible and secreted (extracellular) enzyme (Table 1).

**Table 1:** Growth, organophosphorous phosphatase (OPP) activity and chlorpyrifos degradation

Strain	Growth (OD 610 nm)	OPP activity^a^ (U)		Ratio (*E*/*I*)	Degradation (%)
		Extracellular (*E*)	Intracellular (*I*)		
*L. fermentum*	0.94	0.0068	0.00024	28	70
*L. lactis*	0.59	0.00461	0.00031	15	61
*E. coli*	1.12	0.0032	0.00041	8	16

All strains were grown in MS broth containing CP. OPP and CP degradation was determined after 15 days incubation at 37 °C

One unit (U) is defined as the amount of OPP enzyme liberating 1 μmol *p*-nitrophenol per minute at 37 °C

*U* Unit

^a^Strains grown in CP-lacking MS broth did not show any OPP activity

### Analysis of chlorpyrifos degradation and end product metabolites by LC–MS

Chlorpyrifos is known to be absorbed to surfaces. Nolan et al. (1984) studied the rate of chlorpyrifos absorption by oral administration of pesticide to humans and determined that 70 % was absorbed within 48 h whereas in rats and mice this took a little longer (48–60 h) (Ahdaya et al. 1981). Smith et al. (1967) reported that chlorpyrifos residues were predominantly deposited in fatty tissues. The rate of adsorption, deposition and excretion of chlorpyrifos in humans is a delayed process, and so it should be monitored for a long time to make the study relevant. The optical density and the percentage of chlorpyrifos degraded are given in Table 1. At the end of 15 days, the optical densities of *L. lactis*, *L. fermentum* and *E. coli* were determined to be 0.59, 0.94 and 1.12, respectively, at which time 61, 70 and 16 % chlorpyrifos was degraded, respectively. Interestingly, though *E. coli* had the highest culture density, CP degradation was the lowest. This would suggest that the strains either produced different metabolic end products and/or could tolerate the toxic effects of the same end products to varying degrees.

**Fig. 2:**
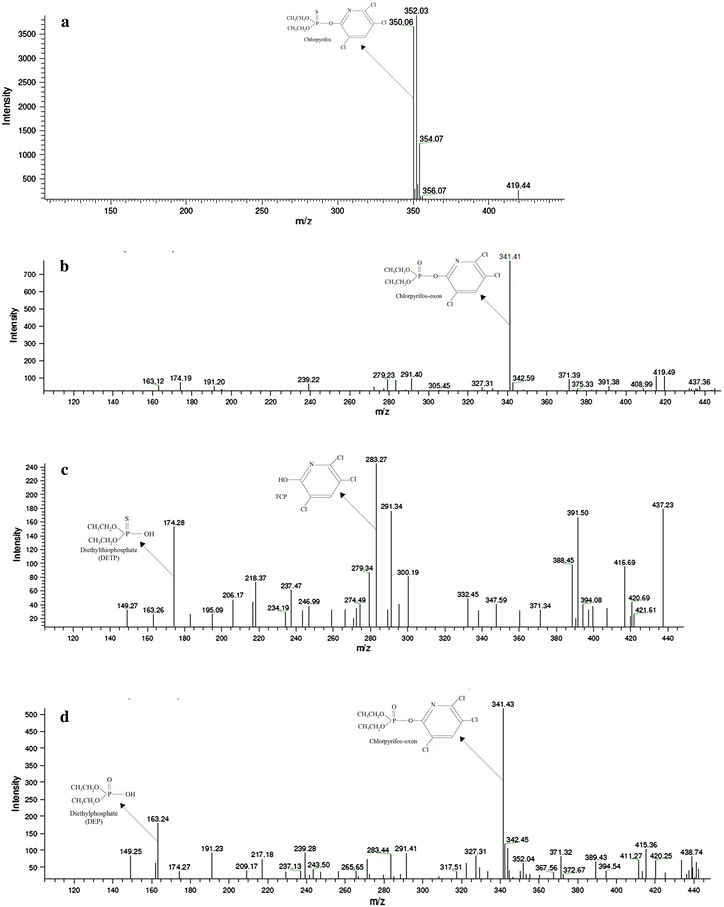
Liquid chromatography–mass spectrometry (LC–MS) spectrum of chlorpyrifos (CP) and degradation products. Mass spectrum of standard chlorpyrifos (100 mg/L) (**a**), mass spectrum of end-products of CP degradation by *L. lactis* (**b**), mass spectrum of end-products of CP degradation by *L. fermentum* (**c**) and mass spectrum of end-products of CP degradation by *E. coli* (**d**). The three strains were grown in minimal salt medium amended with 100 mg/L of chlorpyrifos (CP) at 37 °C for 15 days. CP and the end products of CP degradation were extracted as described in “Materials and methods”

The concentration on CP was determined using LC–MS and a spectrum of chlorpyrifos standard showing a peak with an *m*/*z* value of 350.06 (Fig. 2a). Further LC–MS analysis revealed that each of the three strains produced different metabolic end products from chlorpyrifos degradation. Extract of chlorpyrifos treated with *L. lactis* had a mass value of 341.41, which was similar to *m*/*z* value of chlorpyrifos-oxon (Fig. 2b). Mutch and Williams (2006) had reported that chlorpyrifos is metabolized to chlorpyrifos-oxon by cytochrome P450 enzymes in human liver and could be analogous to that of *L. lactis*. Major and minor peaks of CP degradation by *E. coli* had *m*/*z* values of 341.43 and 163.24 (Fig. 2d) and were identified as chlorpyrifos-oxon and diethylphosphate, respectively. Similarly, *L. fermentum* had three major peaks with *m*/*z* values of 283.27, 291.34 and 174.28, and the peaks identified 3,5,6-trichloro-2-pyridinol (TCP), TCP moiety and diethylthiophosphate (Fig. 2c). A *Sphingomonas* sp. isolated from a polluted water treatment plant and which utilized CP as the sole source of carbon for growth has been reported to produce TCP as an end product (Li et al. 2007).

Xu et al. (2010) had showed that the intestinal microorganisms were efficient in degrading toxic food grade sudan dyes and our studies reported here have extended this to include CP. Our present studies have shown that *L. lactis*, *L. fermentum* and *E. coli* isolates from the gastrointestinal tract can degrade CP. The end product degradation profiles and/or the types of end products are different in all the three strains, which suggest that different pathways may be operating in these strains. The individual role of bacterial strains on chlorpyrifos in the in vitro condition was analysed, but the role in the in vivo as well as efficiency of the strains in consortium needs to investigate. Future studies should also target CP degradation by other gut microbes and also focus on the possible synergistic mineralisation of the end products by other gut microbes.
